# Modeling cooperating micro-organisms in antibiotic environment

**DOI:** 10.1371/journal.pone.0190037

**Published:** 2017-12-28

**Authors:** Gilad Book, Colin Ingham, Gil Ariel

**Affiliations:** 1 Department of Mathematics, Bar-Ilan University, Ramat Gan, Israel; 2 Hoekmine BV, Utrecht, The Netherlands; Beijing Institute of Microbiology and Epidemiology, CHINA

## Abstract

Recent experiments with the bacteria *Paenibacillus vortex* reveal a remarkable strategy enabling it to cope with antibiotics by cooperating with a different bacterium—*Escherichia coli*. While *P*. *vortex* is a highly effective swarmer, it is sensitive to the antibiotic ampicillin. On the other hand, *E*. *coli* can degrade ampicillin but is non-motile when grown on high agar percentages. The two bacterial species form a shared colony in which *E*. *coli* is transported by *P*. *vortex* and *E*. *coli* detoxifies the ampicillin. The paper presents a simplified model, consisting of coupled reaction-diffusion equations, describing the development of ring patterns in the shared colony. Our results demonstrate some of the possible cooperative movement strategies bacteria utilize in order to survive harsh conditions. In addition, we explore the behavior of mixed colonies under new conditions such as antibiotic gradients, synchronization between colonies and possible dynamics of a 3-species system including *P*. *vortex*, *E*. *coli* and a carbon producing algae that provides nutrients under illuminated, nutrient poor conditions. The derived model was able to simulate an asymmetric relationship between two or three micro-organisms where cooperation is required for survival. Computationally, in order to avoid numerical artifacts due to symmetries within the discretizing grid, the model was solved using a second order Vectorizable Random Lattices method, which is developed as a finite volume scheme on a random grid.

## Introduction

Cooperation between bacterial species is an exciting demonstration of how micro-organisms can facilitate dispersal by forming multispecies swarms with mutual benefits [1]. For example, experiments show that different bacterial species can use collective migration to cross barriers and reach new habitats [2–6]. From a modeling point of view, multispecies swarms and colonies present new theoretical and computational challenges, in particular due to a rich phase diagram with a multitude of possible emerging patterns.

**Fig 1:**
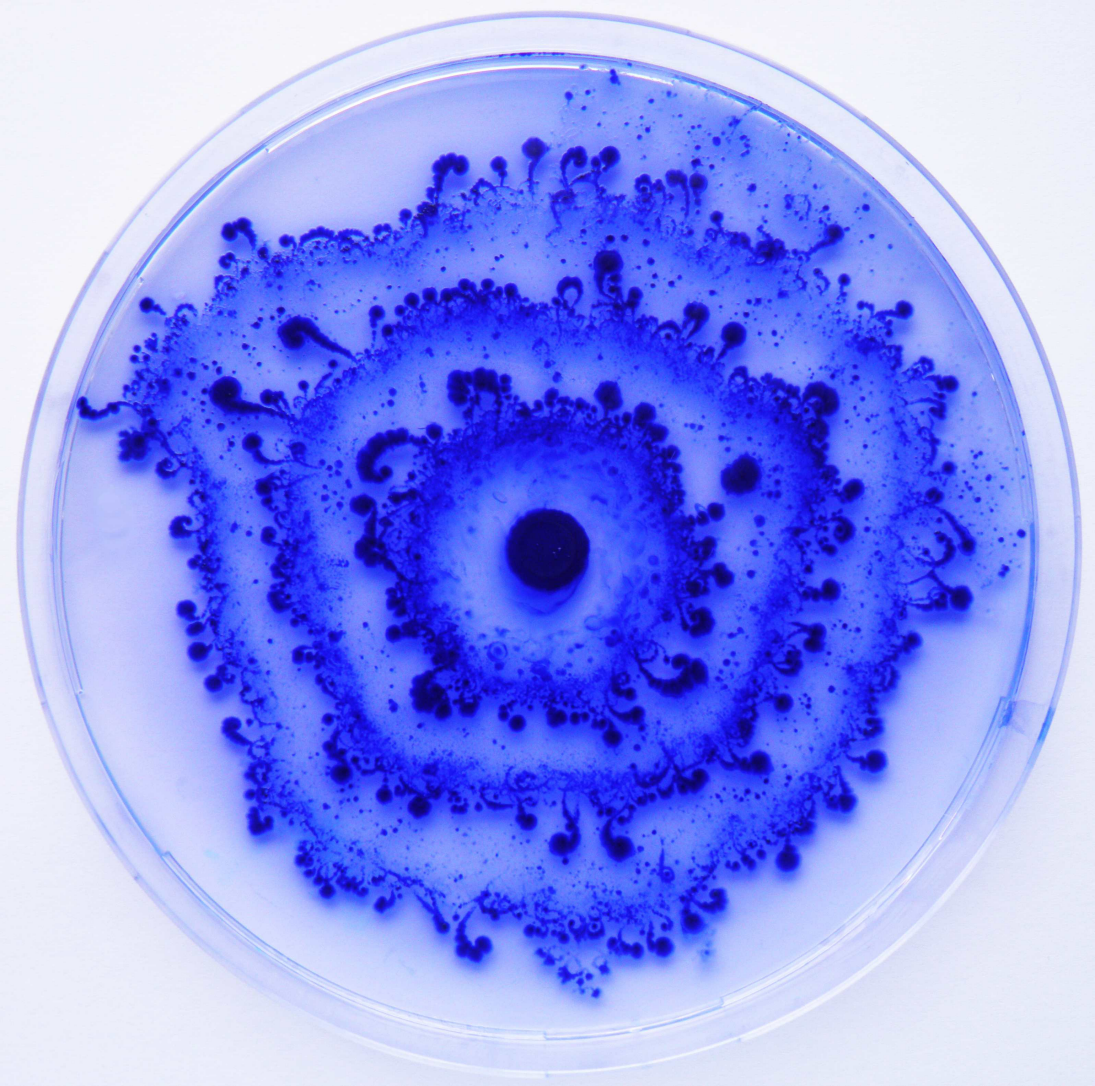
A typical ringed pattern of a mixed *P*. *vortex* and *E*. *coli* bacterial colony on a 14 cm agar plate containing the antibiotic ampicillin. The rings represent different bacterial densities in alternating behaviors of building and expansion. Reproduced from [7].

Here, we focus on explaining recent experiments by Finkelshtein et al. [7] in which the bacterial species *Paenibacillus vortex* (*P*. *vortex*) and *Escherichia coli* (*E*. *coli*) were shown to cooperate in order to survive and grow in an antibiotic-rich environment. It is known that *P*. *vortex* is a highly effective swarmer that can move rapidly over surfaces [8] and move towards nutrients [9] or away from antibiotics [2, 10]. *P*. *vortex* is sensitive to high concentrations of ampicillin, a beta-lactam antibiotic that damages the cell wall, leading to death [7]. In contrast, *E*. *coli* cannot produce the lubricant needed for efficient movement across surfaces on high-percentage agar plates that permit *P*. *vortex* swarming. However, the strain studied carries the gene encoding a beta-lactamase enzyme that can degrade ampicillin [7]. Surprisingly, Finkelshtein et al. [7] show that a mixed colony of *P*. *vortex* and *E*. *coli* can expand the colony size and grow even at high antibiotic concentrations in which neither species can grow on its own—*P*. *vortex* alone dies because of antibiotics while *E*. *coli* alone cannot move toward other regions on the plate when nutrients available locally are completely consumed. The mixed colony develops a ring-shaped pattern with intermittent switching between low and high bacterial densities (see Fig 1). It has been hypothesized that *P*. *vortex* carry *E*. *coli* towards the edge of the colony so that the latter can inactivate the antibiotics. Once the antibiotic has been locally deactivated, the colony continues its expansion. Thus, the ringed pattern is formed by sequential transitions between two growth states: the first is a high-density, cooperating (or expanding) state in which the colony expands rapidly [7], while the second is a low-density, competing (or building) state, in which the different types of bacteria compete for the limited nutrient supply. This two-species cooperation changes the usual, continuous expansion of a *P*. *vortex* colony observed under rich conditions, into a periodic, oscillating growth dynamics.

Following the experimental observations, one of the main assumptions underlying our model is that *P*. *vortex* can appear in two forms or phases [10]. The first, termed a “builder” phase, occurs when the bacterial density is relatively low. Since movement depends on the ability of bacteria to extract liquids from the substrate, motility in this phase is low and depends on the local bacterial density. On the other hand, when the bacterial population is large enough to support the colony’s expansion then the cells transition to a morphologically different phase. This subpopulation, termed “explorer”, is characterized by cell elongation and rapid movement into new territories [10]. We assume that transitions between the phases is a reversible global event that encompasses the entire colony once the average bacterial concentration reaches a critical high or low threshold. See the methods section for details on how these observations are implemented.

In this paper, we present a mathematical model describing the bacterial density for the two bacterial species in an antibiotic environment. Our results are compared with experimental observations. In particular, we confirm the biological hypotheses on which the model is based and shed light on the way in which different bacterial species cooperate in order to survive and develop a shared colony in a hostile environment.

The literature shows a wide variety of approaches to model development of bacterial colonies [1–5,9,11–24]. The proposed models can be divided into two main categories: discrete models [3,4,11,15,19,22] and continuous models [3–5,9,11–21,23,24]. In the discrete approach, the characteristics of individuals or groups in a colony are described, for instance, the location of individuals, nutrient consumption rates and mortality rates. The advantage of these models is the differentiation between the characteristics of different individuals and the ability to follow an individual or specific group for a length of time. However, simulations of realistic bacteria numbers in a colony are not possible. On the other hand, continuous models describe characteristics shared by a large number of cells, for example, local density averages, mean nutrient consumption rates etc. The advantage of these models is their relative simplicity. However, they are not able to focus on a certain bacterial group and the long-term processes influencing it.

A special emphasis was given to the *Paenibacillus* bacteria, which were found to develop unique patterns, branching out from the center of the colony in different directions [17, 18]. Experiments have shown that different pattern characteristics, for example, the number and density of branches and the growth rate, depend on the environment: the growth medium, humidity, temperature, the amount of nutrients and the initial bacterial concentration [20,21]. We will focus and generalize the continuous model suggested by Schwarcz et al. [23], involving coupled partial differential equations. This two-dimensional model was shown to successfully reproduce all possible patterns observed for different colonies and with realistic growth rates. See also [12,13,19,24] for similar approaches.

**Fig 2:**
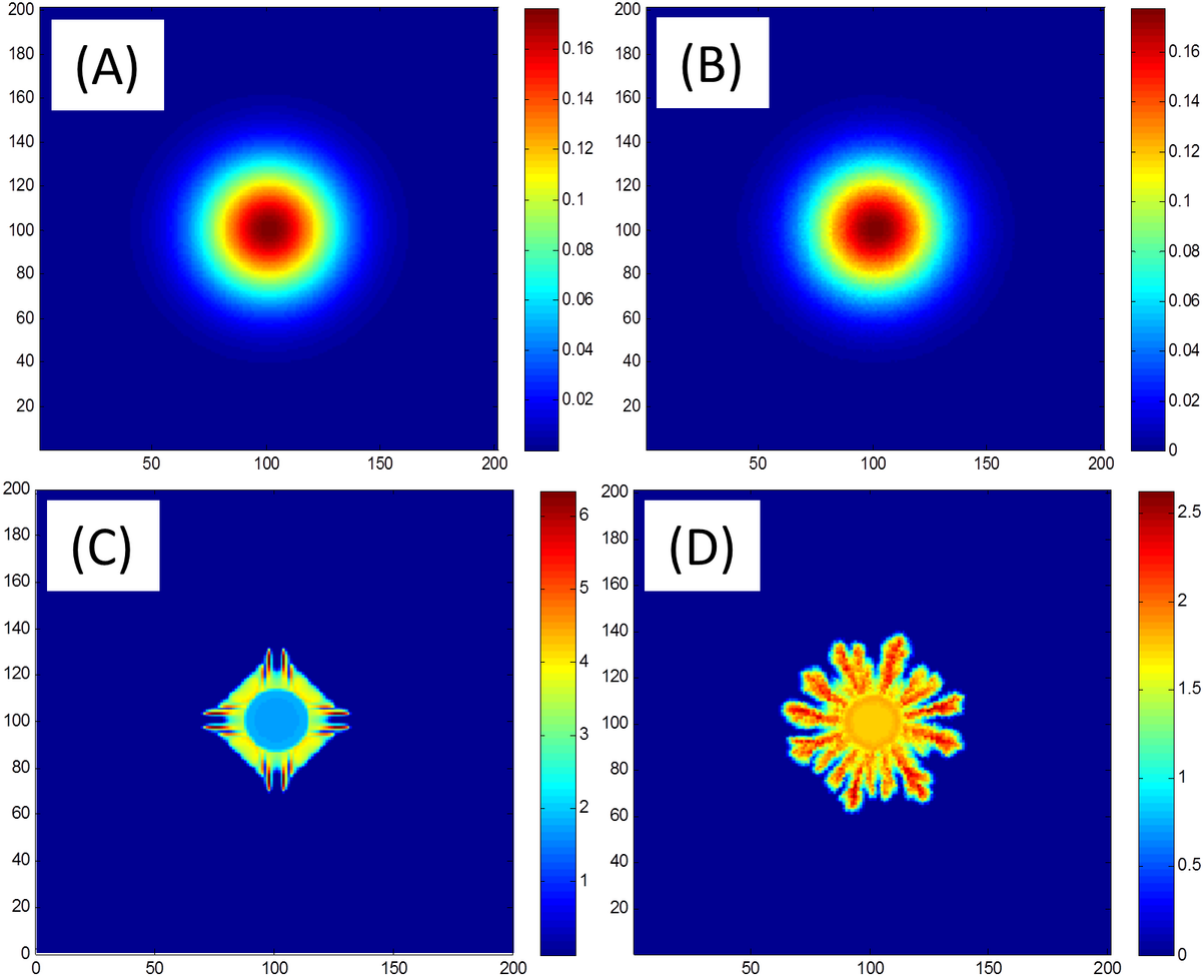
Grid effects. Numerical solutions for the linear (*k* = 0, top) and non-linear (*k* = 1, bottom) diffusion equation ∂*b*/∂*t* = ∇∙(*b*^*k*^∇*b*). With linear diffusion, a rectangular lattice (left) and a random lattice (right) yield similar results. However, with non-linear diffusion, the solution is compact and different lattices yield observably different numerical solutions. In particular, the 4-fold symmetry of the rectangular grid is apparent in figure (C).

One of the main computational challenges in simulating the growth of bacterial colonies and other complicated reaction-diffusion processes showing pattern formation, is that solutions (or solution interfaces) may become unstable. As a result, the numerical methods and, in particular, the grid used for discretization is highly important, as symmetries in the underlying computational lattice can induce similar symmetries in the solution. To overcome this difficulty, we applied a Vectorizable Random Lattice (VRL), in which vertices are a set of randomly chosen points with uniform distribution. See Fig 2 for a simple numerical example. The method, originally proposed in [25], was adapted for simulating the growth of bacterial colonies in [23]. Here, the numerical scheme is re-derived using a finite-volume approach [26], which facilitates generalization to different time-stepping schemes and conservative high-order methods.

## Materials and methods

For a single species, the model is described in terms of a two-dimensional system of reaction-diffusion equations as follows (see also [12,13,19,23,24]).

$$\begin{array}{l} \frac{\partial b}{\partial t}=\nabla (D_{b}b^{k}\nabla b)+\beta (n)b-\mu (n)b\\ \frac{\partial n}{\partial t}=D_{n}\Delta n-\lambda (n)b \end{array}$$ (1)

- The first Eq describes the density of bacteria *b*(*x*,*y*,*t*). The first term is a nonlinear diffusion with a coefficient that depends on *b*. The exponent *k* describes the mobility of bacteria within the medium [12,13]. Following [23], we take *k* = 0 for explorers and *k* = 1 for builders (see details below). The second and third terms describe the bacterial reproduction and death rates, which may depend on the local nutrient concentration [27].
- The second Eq describes the nutrient density *n*(*x*,*y*,*t*), where, the first term is diffusion and the second describes nutrient consumption.

For the rest of the paper, bacteria densities is measured in terms of the local area fraction that cells occupy.

The main purpose of the paper is to generalize the model (1) to the two-species case with antibiotics.

$$\begin{array}{l} \frac{\partial b_{1}}{\partial t}=\nabla \left[D_{1}b_{1}^{k}\nabla b_{1}\right]+\beta_{1}(n)b_{1}-\mu_{1}(n,a)b_{1}\\ \frac{\partial b_{2}}{\partial t}=\nabla \left[D_{2}\nabla b_{2}\right]+\nabla \left[C_{2}b_{2}\nabla b_{1}\right]+\beta_{2}(n)b_{2}-\mu_{2}(n)b_{2}\\ \frac{\partial n}{\partial t}=\nabla \left[D_{n}\nabla n\right]-\lambda_{1}(n)b_{1}-\lambda_{2}(n)b_{2}\\ \frac{\partial a}{\partial t}=\nabla \left[D_{a}\nabla a\right]-p(a)b_{2} \end{array}$$ (2)

- The first Eq. describes the density of *P*. *vortex*, denoted *b*_1_(*x*,*y*,*t*). This equation is similar in form to the equation presented in model (1), except that the death term *μ*_1_(*n*,*a*)*b*_1_ depends also on the antibiotics concentration, *a*(*x*,*y*,*t*).
- The second Eq. describes the density of *E*. *coli*, denoted *b*_2_(*x*,*y*,*t*). The first term describes the (low) self-movement of *E*. *coli* as a linear diffusion with a small parameter, *D*_2_ ≪ *D*_1_. The second term describes advection with the flow of *P*. *vortex*, i.e., in the direction of ∇*b*_1_. This component is the main movement component for *b*_2_, i.e., *D*_2_ ≪ *C*_2_. The last two terms are reproduction and death. Note that *E*. *Coli* is not sensitive to the antibiotics used in the experiments and, accordingly, its death rate does not depend on *a*.
- The third Eq. describes the nutrient concentration *n*(*x*,*y*,*t*), similar to (1b).
- The last Eq describes the antibiotic concentration *a*(*x*,*y*,*t*), where, the first term describes diffusion and the second term describes the inactivation of the antibiotics by *E*. *coli*.

Following [23], the mortality rates are taken as, *μ*_1_(*n*,*a*) = 0.3*a*/(1 + 4*n*) and *μ*_2_(*n*) = 1/(1 + 4*n*). Reproduction, nutrients and antibiotics degradation rates are taken as linear functions. See Table 1 for simulation values and [23] for a detailed discussion on the relation between simulated and physical magnitudes.

**Table 1:** The different parameter values used in simulations. Conversions between simulated and physical units are discussed in the results section.

Parameter	Value	Description
Δ*t*	0.001	Time step length
*D*_1_(exploring)	0.35	Diffusion coefficient of *P*. *vortex* at exploring state
*D*_1_(building)	0.0125	Diffusion coefficient of *P*. *vortex* at building state
*D*_2_	0.0001	Self-motility diffusion coefficient of *E*. *coli*
*C*_2_	6	Advection coefficient of *E*. *coli* with the flow of *P*. *vortex*
*D*_*n*_	0.25	Diffusion coefficient of the nutrients
*D*_*a*_	0.25	Diffusion coefficient of the antibiotics
*β*_1_(exploring)	0.7	*P*. *vortex* reproduction rate at exploring state
*β*_1_(building)	0.9	*P*. *vortex* reproduction rate at building state
*β*_2_	0.5	*E*. *coli* reproduction rate
*k*(exploring)	0	Linear diffusion of *P*. *vortex* at exploring state
*k*(building)	1	Linear diffusion of *P*. *vortex* at building state
*λ*_1_	0.9	Nutrients consumption rate by *P*. *vortex*
*λ*_2_	0.9	Nutrients consumption rate by *E*. *coli*
*n*_0_	2	Initial concentration of *n*
*p*	0.6	Antibiotic decomposition rate
*a*_0_	2	Initial concentration of *a*
${\bar{b}}_{\max}$	0.033	Maximal value for *b*_*1*_ at building state
${\bar{b}}_{\min}$	0.007	Minimal value for *b*_*1*_ at exploring state
*r*_0_	0.15 × the domain size	Initial colony radius

We assume no-flux conditions on the domain boundary. In experiments, at the initial inoculation spot the two species are homogenously mixed (normally 50:50). The next stage is the formation of swarming groups of P. vortex but the system rapidly (24 h) corrects to approximately 50:50 even if the initial inoculation is not balanced. The antibiotic and nutrients are initially distributed homogeneously. Accordingly, nutrient and antibiotics initial concentrations are constants, denoted *n*_0_ and *a*_0_. The initial bacterial concentrations are a smoothed circle (see below).

Following the biological considerations described above, transitions between the exploring and building states depend on the average density of *P*. *vortex* bacteria in the entire colony,

$${\bar{b}}_{1}=\frac{\int_{B}b_{1}dA}{\int_{B}dA},\;\mathrm{where}\;B=\left\{(x,y)|b_{1}(x,y)>10^{-6}\right\}$$ (3)

Initially, all bacteria are builders. In this phase, motility depends on the ability of cells to extract liquids from the substrate which, in turn, depends on the local bacterial density. Hence, we take *k* = 1. It has been shown that with a nonlinear diffusion, the colony’s boundary is sharp, which is consistent with the biological picture of a relatively stationary growth stage. Once ${\bar{b}}_{1}$ becomes larger than a prescribed threshold ${\bar{b}}_{\max}$, the entire colony transitions to a new state of explorers in which *k* = 0. In this phase, the expansion rate of the colony is significantly larger. As a result, the average bacterial density reduces. The colony will switch back to a building state if ${\bar{b}}_{1}$ becomes smaller than a second threshold ${\bar{b}}_{\min}$. Selected parameters values are detailed in Table 1.

The value of ${\bar{b}}_{\max}$ also sets the physical scale for the bacterial concentrations. Assuming that ${\bar{b}}_{\max}$ corresponds to an area fraction of approximately 50%, then, unit concentration *b*_*i*_ = 1 corresponds to an area fraction of about $1/2{\bar{b}}_{\max}$.

### The numerical method

As discussed above, one of the main difficulties in solving equations of the form of (1) and (2) is that symmetries in the discretizing lattice may become manifested in the numerical solution [16,23,24]. Different approaches have been suggested to overcome this problem. For example, [16] suggest adding some low-amplitude random noise to solutions on regular lattices. Instead, we will apply a random grid method, which does not exhibit any artifact symmetries. We will follow the Vectorizable Random Lattice (VRL) approach developed in [23,25]. One of the main goals of this paper is to further develop the numerical analysis of nonlinear reaction-diffusion equations on the VRL.

The random lattice was first presented by Christ et al. [28]. Its main feature is the random selection of points in the domain. According to [28], the number of vertices on a random lattice in a given area or volume is a random variable with a Poisson distribution (which is called a Poisson random lattice). The randomness decreases the measurable influence of the lattice on the numerical solution and increases isotropy.

Despite the significant advantage of the Poisson random lattice, its use is numerically complex. In particular, the use of efficient numerical methods such as implicit schemes or implementing parallel computation is difficult [29–32]. In order to take advantage of the random lattice on one hand, but enable the development of efficient numerical methods on the other, Moukarzel and Hermann (1992) suggested the VRL [25]. The VRL is also composed of random nodes, however, as opposed to the Poisson Random Lattice, the nodes are drawn in a particular way, giving it additional structure. See Appendix A for further details.

## Results

The model (2) was solved with various initial conditions for the functions *b*_1_ and *b*_2_, reproducing different experimental setups. Accordingly, the simulated domain corresponds to 90 mm and every simulation time unit corresponds to about 0.5 hours. For example, units for diffusion constants are about 1.5∙10^−6^m/sec^2^ (recall densities are measured in terms of area fraction).

**Fig 3:**
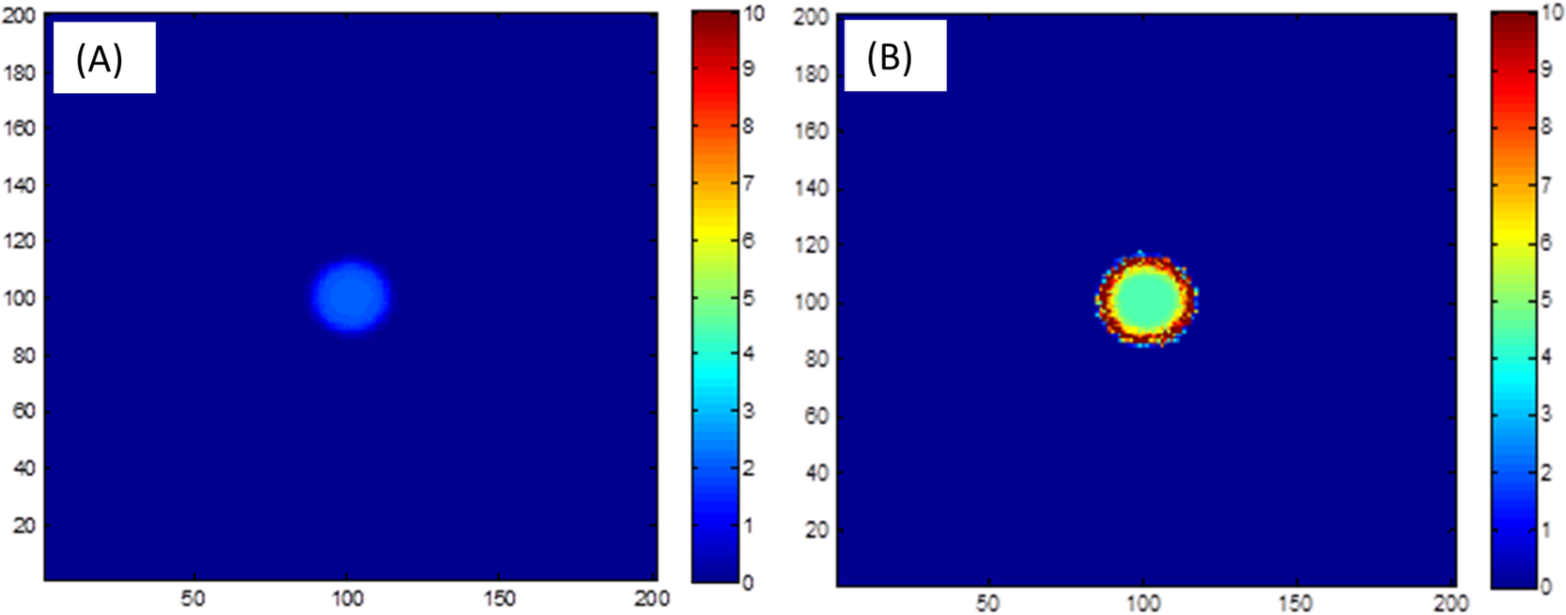
Single-species simulations. Individual species cannot grow. (A) On its own, *P*. *vortex* dies due to antibiotics and the colony does not expand. (B) On its own, *E*. *coli* does not expand because the bacteria are unable to move independently towards a nutrient rich area. Simulation parameters are detailed in Table 1. Simulation time is equivalent to about 50 hours.

First, we consider each species on its own, i.e., either *b*_1_ = 0 and $b_{2}=1_{r<r_{0}}$ or the other way around. The function $1_{r<r_{0}}$ is a smoothed (twice differentiable) indicator function of the unit disk {*r* < *r*_0_}. Fig 3 shows that, as seen in experiments, neither *P*. *vortex* nor *E*. *coli* can grow on its own–a colony of *E*. *coli* cannot expand because of its low motility, while *P*. *vortex* dies due to antibiotics. In order to depict the colony and its history, the figure shows the accumulated bacterial density *s*_*i*_ + *b*_*i*_, where *ds*_*i*_/*dt* = *μ*_*i*_*b*_*i*_, i.e., the sum of live and dead bacteria at location (*x*,*y*) up to time *t*.

**Fig 4:**
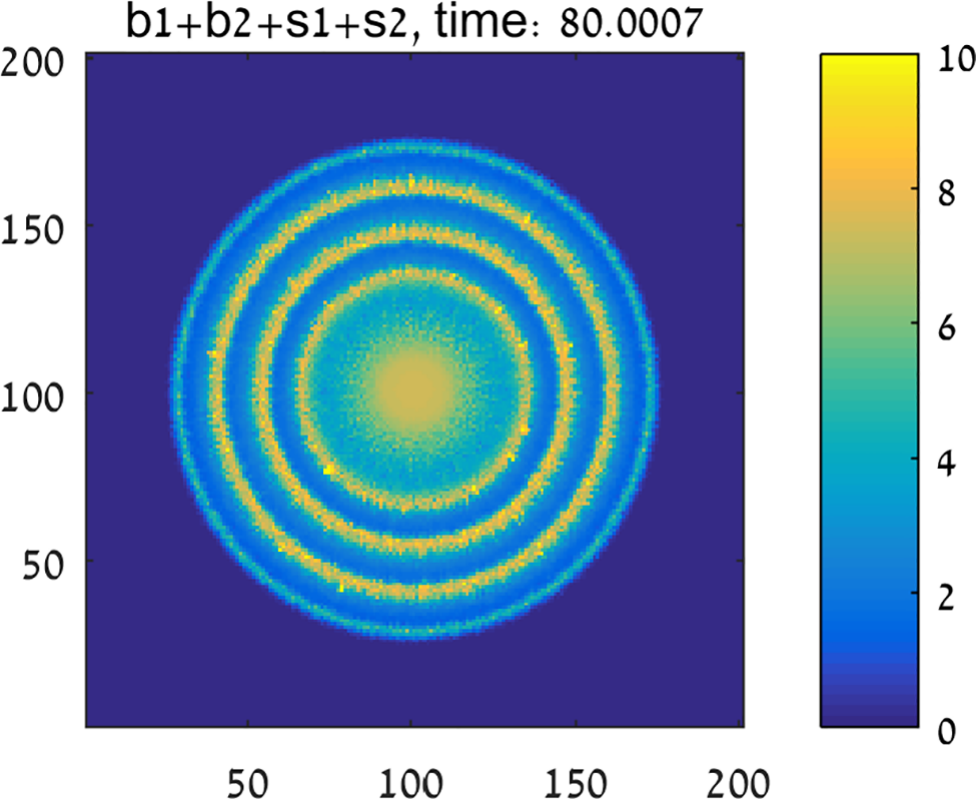
Two-species simulations. The joint *P*. *vortex* and *E*. *coli* bacterial colony in an antibiotic environment develops ring-like patterns. All simulation parameters are the same as in Fig 3.

**Fig 5:**
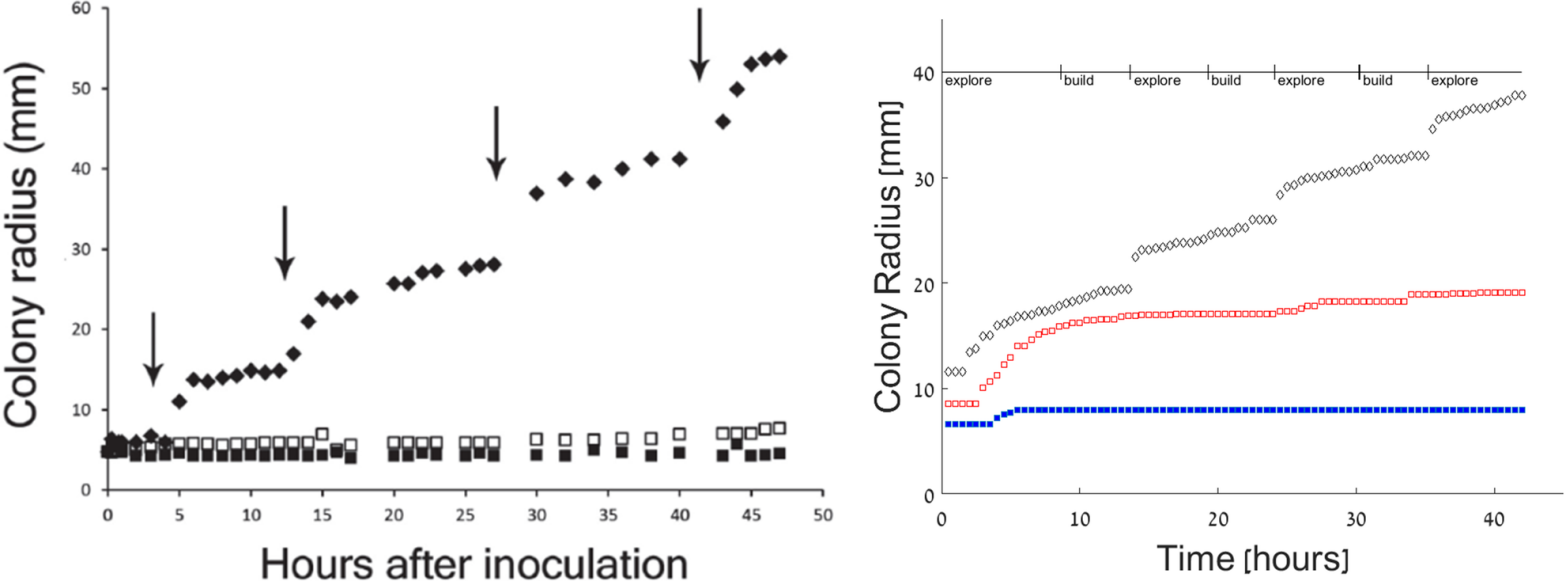
Comparing experiments and simulations. The colony radius as a function of time. Left: experiments (reproduced from [7] showing *P*. *vortex* alone (full blue squares), *E*. *coli* alone (empty red squares) and the combined colony (black diamonds). Right: Simulations. Both figures show the non-continuous increase in the radius of the joint colony but only small, marginal expansion of each species on its own. Simulation units were converted to experimental ones as explained in the text.

**Fig 6:**
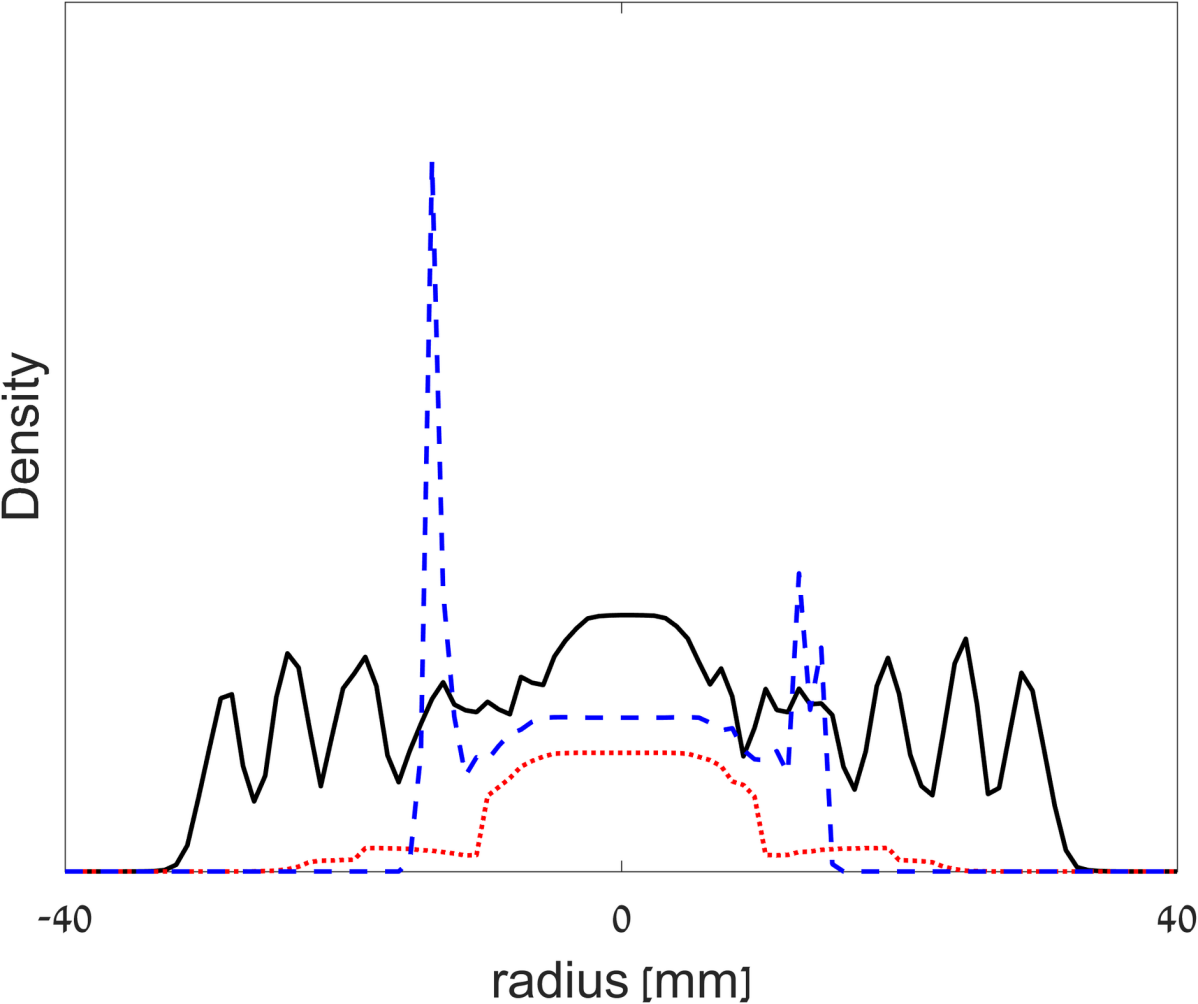
A snapshot of the simulated cross-sections of the different colonies: *P*. *vortex* and *E*. *coli* (solid black line), only *P*. *vortex* (dotted red) and only *E*. *coli* (dashed blue).

In contrast to these results, Fig 4 shows that a mixed colony (initial conditions $b_{1}=b_{2}=1_{r<r_{0}}$) can grow successfully, showing a ring-like pattern, qualitatively similar to experiments. The rings are of intermittently high and low density and each one represents a different state of the *P*. *vortex* bacteria–exploring or building. Fig 5 depicts the radius of the colonies in all three cases described above. Similar to the experimental observations (Fig 5A), the simulated colony (Fig 5B) shows a non-continuous expansion rate, corresponding to the alternating building and exploring states. Fig 6 shows a cross section of the colonies. While the cross section of the mutual colony is periodic across the radius, *P*. *vortex* alone shows a low density decreasing towards the edge (due to antibiotic). On the other hand, the density of *E*. *coli* increases towards the edge, where nutrients are higher.

It is interesting to note that the periodicity is in the radius rather than the area. This is in accordance with theories describing the advancing edge as a traveling wave propagating at constant speed along the colony’s radius [1–5,9,11–24,33–35]. Our simulations suggest that the builder/explorer phases expand with distinct wave propagation speeds and that the transitions are entrained to changes in the concentration of antibiotics at the front.

In addition, we note that the results are not specific to the parameters detailed in Table 1. See S1 Fig for a qualitative description of the dependence of the results on different parameters. Another possible variation of our model is to assume that the builder/explorer transition depends on the average density of both *P*. *vortex* and *E*. *coli*. To this end, Eq (3) was modified by replacing *b*_1_ with *b*_1_ + *b*_2_. S2 Fig show that our results are practically unchanged. All simulation parameters are the same, except for slightly shifted values for ${\bar{b}}_{\min}$ and ${\bar{b}}_{\max}$ (0.029 and 0.066, respectively).

**Fig 7:**
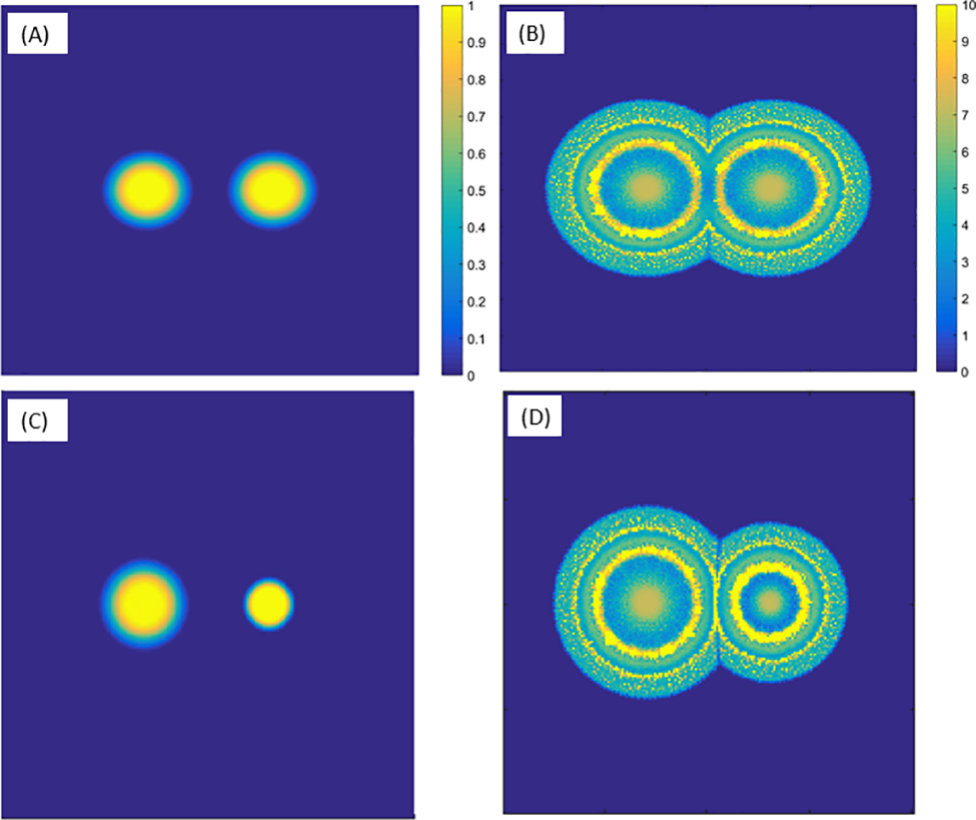
Simulation results with two colonies (*P*. *vortex*+*E*. *coli*). Left: Initial colonies. Right: After 30 hrs. Upon contact, the builder/explorer phases of the two colonies synchronize.

In order to further examine the coupling between the builder/explorer transitions with the multi-species interaction, we study the dynamics of our model under initial conditions which were not realized in the experiments of [7]. Fig 7 depicts simulation results with initial two separated colonies, each one containing both *P*. *vortex* and *E*. *coli*. While separated, each colony determines the builder/explorer phase on its own. Once the two colonies overlap, the phase is the same and the colonies synchronize.

**Fig 8:**
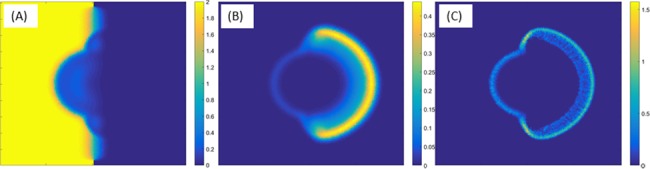
Simulation results for a plate in which only the left half of the domain initially has antibiotics. (A) The concentration of antibiotics, (B) *P*. *vortex* and (C) *E*. *coli* after 13hrs. The colony only grows to the right (no antibiotic) although in principle, the mixed colony can also grow to the left.

Fig 8 depicts simulation results with an initial sharp gradient in the antibiotic concentration, *a*(*x*,*y*,*t*) = 1_{*x*<0}_, i.e., only the left half of the domain contains antibiotics. All other parameters are exactly the same. Interestingly, we see that although the mixed colony can expand in all directions, the colony only grows towards the antibiotics-free half.

**Fig 9:**
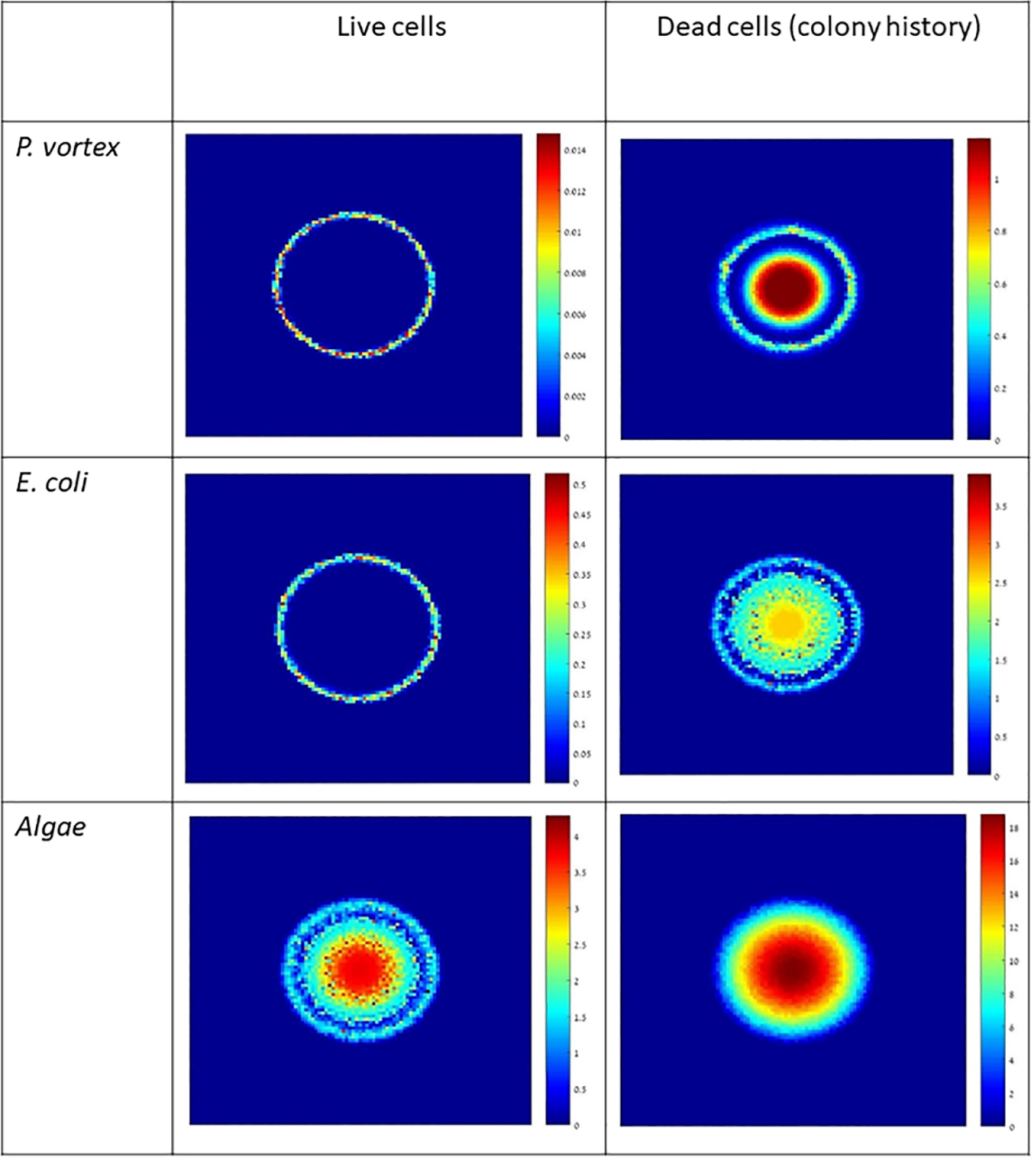
Simulation results for a 3-species system.

Finally, we begin to explore more complicated systems and dynamics. We consider a joint *P*. *vortex* and *E*. *coli* colony that also includes a microalgae, such as *Chlorella vulgaris*. This algae is non-motile but can produce carbon that acts as additional nutrients to the bacteria (Finkelshtein and Polikovsky, unpublished). It has been suggested that *P*. *vortex* can transport and use the algae to fix carbon. See Appendix B for the precise formulation of the model and simulation details. Fig 9 depicts simulation results showing that *P*. *vortex* carries both micro-organisms. Our preliminary results show that not only is such a system stable, it can facilitate a more rapid colony expansion than in the absence of *C*. *vulgaris*.

### Discussion

In nature, bacterial cultures are typically highly heterogenous, involving thousands of bacterial species and other micro-organisms. Within such diverse systems, cooperation is a major survival strategy. This includes cooperative movement (e.g. swarming) in multi-species suspensions [1]. Taking the first steps in understanding such cultures, several recent experiments considered simple mixtures of 2 or 3 micro-organisms, for example, two bacteria or bacteria and fungi, revealing highly complex dynamics and patterns.

Here, we present a model describing cooperation between *P*. *vortex* and *E*. *coli* species. The model describes the joint movement of bacteria in a hostile environment containing antibiotics and sparse nutrients. Our results demonstrate that, on one hand, the bacteria must cooperate in order to survive this environment while at the same time compete over the available nutrients. The model was solved on a Vectorizable Random Lattice, using new high order schemes that conserve mass. Similar to experiments, simulations show a ring-like pattern consistent with intermittent builder and explorer bacterial phases.

Our exploration into new possible system setups offer several predictions that may be tested experimentally. For example, with a sharp antibiotics gradient, our simulations suggest that the mixed colony only grows towards the low-antibiotics region. This occurs although our model does not include explicit terms that enable *P*. *vortex* to “unload” *E*. *coli* if it is not needed (to save energy). Simulations with two colonies suggest that the ring pattern should synchronize once the colonies are in contact. Such an experiment can validate our assumption that that builder/explorer phase is a global colony-wide property.

Finally, to the best of our knowledge, our three-species model is the first attempt to model realistic three-species colonies including phenotype heterogeneity (*P*. *vortex* builder/explorers) with a particular function or objective in mind. An additional important factor, which is not currently taken into account in our model, is the ability of *P*. *vortex* to deposit, or unload *E*. *coli* or algae. This is particularly important if the larger algae could overgrow. For example, this could be modelled as an upper limit on transport capacity.

Understanding the dynamics of multi-species colonies is a rapidly growing field as microbiologist are increasingly aware of swarming organisms composed of multiple subpopulations [36]. This work takes another step towards simulating a 'moving ecosystem' or the construction of ‘multispecies consortia’ within the growing field of synthetic ecology [37].

## Appendix A: The numerical method

In this appendix, we review the construction of the Vectorial Random Lattice (VRL). In addition, we present a new systematic method for discretizing nonlinear reaction-diffusion equations such as (1) or (2) using the finite volume approach [26]. We apply both forward-Euler and Crank-Nicolson time steps and discuss solution of the resulting implicit equations using iterative methods.

**Fig 10:**
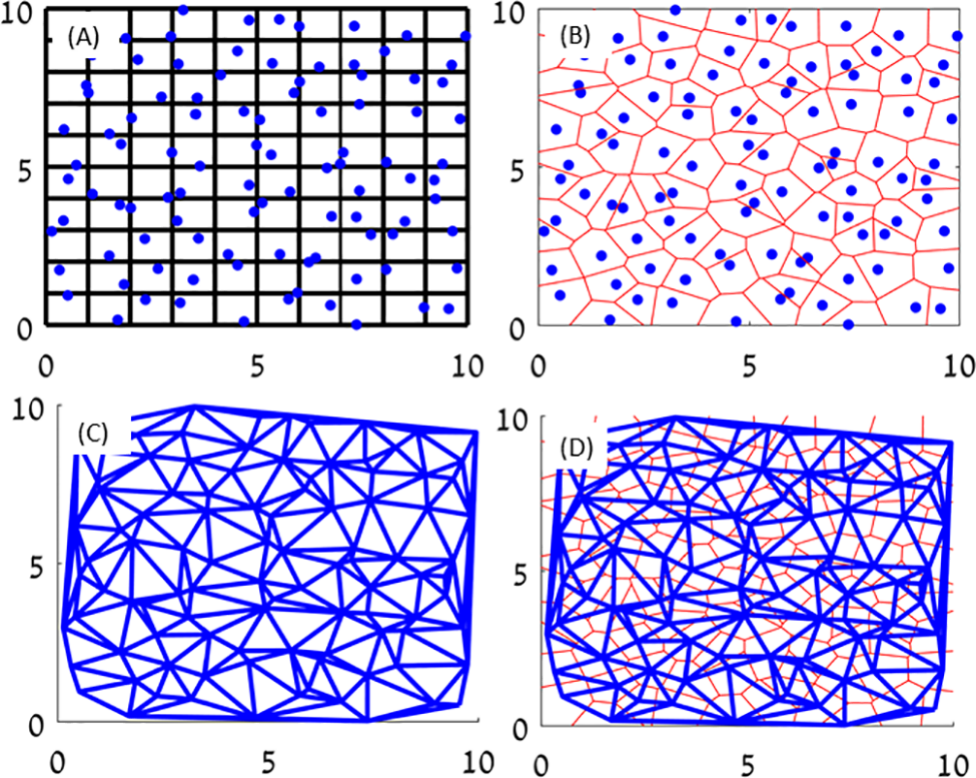
The stages of creating the VRL. (a) A uniform reference lattice with a single node chosen uniformly in each cell. (b) The Voronoi diagram for the random nodes. (c) The Delaunay triangulation yields (d), the final VRL.

The VRL is constructed by first choosing a single point drawn randomly in each cell of a uniform rectangular lattice with grid spacing *h* (called the reference lattice). Let 0 ≤ *d*_*i*_ ≤ *h* denote a minimal distance between two adjacent random nodes in direction *i*. If the distance between two points in cells that are adjacent in the direction *i* are closer than a distance *d*_*i*_, then the two points are redrawn. Therefore, the values of *d*_*i*_ determine the randomness of the lattice: for *d*_*i*_ = 0 randomness is maximal, while with *d*_*i*_ = *h* the lattice is rectangular. The set of random points chosen within the cells of the reference lattice are the nodes of the VRL. Consider the Voronoi diagram associated with this set of points. A pair of nodes {*v*_*i*_,*v*_*j*_} with *i* ≠ *j* are called natural neighbors in the Voronoi partition if *v*_*i*_ and *v*_*j*_ share a common face. The Delaunay triangulation, which is the VRL, is obtained by linking all pairs of natural neighbors. The process is depicted in Fig 10.

An equivalent method for obtaining the Delaunay triangulation for the randomly drawn nodes consists of finding all the trios of nodes located on the perimeter of a circle that does not contain (inside or on the edge) additional nodes. The three nodes will be natural neighbors with each other [30,31,32].

In contrast to uniform lattices, in which all interior nodes have the same number of neighbors, the number of neighbors in VRL varies. The mean number of neighbors is roughly six (identically, the average number of faces in each polygon in a Voronoi diagram is approximately six). The closer two cells on the reference lattice are to each other, the probability that the Voronoi nodes in those cells will be natural neighbors increases. However, there is a non-zero probability that a pair of nodes from non-adjacent cells on the reference lattice are also natural neighbors, thus enabling isotropic connections of the nodes. The probabilities of natural neighbors being found in different distances was calculated in [25]. In particular, it drops sharply (below 10^−4^) for the next-next-nearest neighbors.

### Calculating the Laplacian on a VRL

The Laplacian operator in the diffusion equation is composed of the divergence of the flux of a conserved quantity. In general, for a given flux vector *J*, the diffusion term takes the form,

∇·J=∇·[ψ(x,y,φ)∇φ],

Where *φ*(*x*,*y*) and *ψ*(*x*,*y*,*φ*) are given function. For example, *φ* = *b* and *ψ* = *b*^*k*^ for a nonlinear diffusion, with *k* = 0 for the Laplacian.

**Lemma 1**: Let *φ*(*x*,*y*) denote a continuously twice differentiable function and let *μ*_*i*_ be the set of Voronoi nodes which are natural neighbors of a node *v*_*i*_. Then, the Laplacian is given by

$$\Delta \varphi (v_{i})=\sum_{j\in \mu_{i}}w_{ij}\left[\varphi (v_{j})-\varphi (v_{i})\right]+O(h^{2}),$$

where *w*_*ij*_ = *f*_*ij*_/(*l*_*ij*_*A*_*i*_) and,

- *f*_*ij*_ is the length of the mutual face connecting nodes *v*_*i*_ and *v*_*j*_.
- *l*_*ij*_ is the Euclidean distance between *v*_*i*_ and *v*_*j*_.
- *A*_*i*_ is the area of *V*_*i*_, the Voronoi cell to which *v*_*i*_ belongs.

**Proof:** We will prove lemma 1 using the finite volume approach. See [23,32] for an alternative derivation.

In order to numerically approximate Δ*φ*(*v*_*i*_), we calculate the average value of the Laplacian in each Voronoi cell,

$$\Delta \varphi (v_{i})\approx \bar{\Delta \varphi (v_{i})}=\frac{1}{A_{i}}\int_{V_{i}}\nabla \cdot \nabla \varphi ds=\frac{1}{A_{i}}\int_{\partial V_{i}}\nabla \varphi \cdot \hat{n}dl,$$ (4)

where *ds* is an area element, ∂*V*_*i*_ is the boundary of *V*_*i*_, *dl* a length element, $\hat{n}$ an external normal to *V*_*i*_ and we have used the divergence theorem. For a uniform grid with spacing *h*, the error in approximating Δ*φ* by its average in a cell is of the order *h*^2^ [26]. This is not as simple in the random grid because there is some small probability that the distance to one of the natural neighbors is large. Nonetheless, the probability of such an event decreases rapidly. For example, the probability to have a neighbor with distance larger than 4*h* is smaller than 0.01 [25]. Moreover, for any *ϵ* > 0, there exists a constant *D* independent of *h* such that the probability to have a neighbor with distance larger than *Dh* is smaller than *ϵ*. We therefore neglect this event and assume that the accuracy of the finite volume approximation is of order *h*^2^.

If *v*_*i*_ has *m* natural neighbors, its boundary ∂*V*_*i*_ can be written as the union of *m* polygonial faces. Denoting the common face of the *V*_*i*_ and *V*_*j*_ cells by *K*_*ij*_, the integral on the RHS of Eq (4) can be written as

$$\frac{1}{A_{i}}\sum_{j=1}^{m}\int_{K_{ij}}\nabla \varphi \cdot \hat{n}dl=\frac{1}{A_{i}}\sum_{j=1}^{m}\frac{\varphi_{j}^{n}-\varphi_{i}^{n}}{l_{ij}}f_{ij}+O(h^{2})=\sum_{j=1}^{m}w_{ij}\left(\varphi_{j}^{n}-\varphi_{i}^{n}\right)+O(h^{2}),$$

which concludes the proof of Lemma 1.

Lemma 2:

$$\nabla \cdot [\psi \nabla \varphi ]=\frac{1}{2}\sum_{j\in \mu_{i}}w_{ij}\left[\psi (v_{j})+\psi (v_{i})\right]\left[\varphi (v_{j})-\varphi (v_{i})\right]+O(h^{2}).$$

The proof is similar to Lemma 1.

Using the formulas derived above, different numerical schemes such as forward-Euler, backward-Euler and Crank-Nicolson can be easily developed. For example, with forward-Euler, the numerical flux from cell *V*_*i*_ to cell *V*_*i*_ is given by

$$J_{ji}^{\mathrm{FE}}=\frac{1}{2}w_{ij}\left(\psi_{i}^{n}+\psi_{j}^{n}\right)\left(\varphi_{i}^{n}-\varphi_{j}^{n}\right),$$

where $\varphi_{i}^{n}=\varphi (v_{i},n\Delta t)$. In particular, the flux is skew-symmetric,

$$J_{ji}^{\mathrm{FE}}=-J_{ij}^{\mathrm{FE}},$$

which implies that the discretized concentration *φ* is conserved. Similarly, using Crank-Nicolson, the numerical flux is given by,

$$J_{ji}^{\mathrm{CN}}=\frac{1}{4}w_{ij}\left[\left(\psi_{i}^{n}+\psi_{j}^{n}\right)\left(\varphi_{i}^{n}-\varphi_{j}^{n}\right)+\left(\psi_{i}^{n+1}+\psi_{j}^{n+1}\right)\left(\varphi_{i}^{n+1}-\varphi_{j}^{n+1}\right)\right],$$

which is again skew-symmetric.

### Iterative solutions for implicit methods

In our model, we take *ψ* = *φ*^*k*^, and the Crank-Nicolson scheme reduces to

$$\frac{\varphi_{i}^{n+1}-\varphi_{i}^{n}}{\Delta t}=\frac{D}{4}\left\{\sum_{j\in \mu_{i}}w_{ij}\left[{\left(\varphi_{j}^{n}\right)}^{k}+{\left(\varphi_{i}^{n}\right)}^{k}\right]\left(\varphi_{j}^{n}-\varphi_{i}^{n}\right)+\sum_{j\in \mu_{i}}w_{ij}\left[{\left(\varphi_{j}^{n+1}\right)}^{k}+{\left(\varphi_{i}^{n+1}\right)}^{k}\right]\left(\varphi_{j}^{n+1}-\varphi_{i}^{n+1}\right)\right\}.$$

With linear diffusion, *k* = 0, solving for $\varphi_{i}^{n+1}$ amounts to a sparse linear set of equations. However, with a nonlinear diffusion, *k* ≥ 1, the system becomes non-linear. For simplicity, we consider the case *k* = 1, which is relevant to our model. Larger values of *k* are treated similarly. First, rewrite the Crank-Nicolson scheme as,

$$\varphi_{i}^{n+1}\left(1+\frac{D\Delta t}{4}\sum_{j\in \mu_{i}}w_{ij}\varphi_{i}^{n+1}\right)=\frac{D\Delta t}{4}\sum_{j\in \mu_{i}}w_{ij}\left[{\left(\varphi_{j}^{n}\right)}^{2}-{\left(\varphi_{i}^{n}\right)}^{2}\right]+\frac{D\Delta t}{4}\sum_{j\in \mu_{i}}w_{ij}{\left(\varphi_{j}^{n+1}\right)}^{2}+\varphi_{i}^{n}.$$

Using matrix notation, the system takes the form,

$$\begin{array}{l} \left[I-\frac{D\Delta t}{4}W\times \mathrm{diag}(\Phi^{n+1})+\frac{D\Delta t}{4}Z\times \mathrm{diag}(\Phi^{n+1})\right]\Phi^{n+1}=\\ \left[I+\frac{D\Delta t}{4}W\times \mathrm{diag}(\Phi^{n})-\frac{D\Delta t}{4}Z\times \mathrm{diag}(\Phi^{n})\right]\Phi^{n}, \end{array}$$ (5)

where, assuming a *N* × *N* reference lattice, $\Phi^{n}\in {\mathbb{R}}^{N^{2}}$ denotes the solution vector

$$\Phi^{n}={\left(\varphi_{v_{1}},\ldots ,\varphi_{v_{N^{2}}}\right)}^{T},$$

$W\in M_{N^{2}}$ denotes the *N*^2^ × *N*^2^ weighted adjacency matrix,

$$W_{ij}=\{\begin{array}{cc} w_{ij} & j\in \mu_{i}\\ 0 & j\notin \mu_{i} \end{array}$$

and $Z\in M_{N^{2}}$ is a diagonal matrix with elements $Z_{ii}=\sum_{j\in \mu_{i}}w_{ij}$. In the above, diag(Φ^*n*^) denotes the *N*^2^ × *N*^2^ diagonal matrix with Φ^*n*^ on the diagonal. Noting that all matrices are sparse, the system (5) is solved iteratively using a splitting method (we applied Gauss-Seidel). For each *n*, we formally write,

$$\begin{array}{l} \left[I-\frac{D\Delta t}{4}W\times \mathrm{diag}(V^{\left(r\right)})+\frac{D\Delta t}{4}Z\times \mathrm{diag}(V^{\left(r\right)})\right]\Phi^{n+1}=\\ \left[I+\frac{D\Delta t}{4}W\times \mathrm{diag}(\Phi^{n})-\frac{D\Delta t}{4}Z\times \mathrm{diag}(\Phi^{n})\right]\Phi^{n} \end{array}$$

and begin with an initial guess, *V*^(0)^ = Φ^*n*^. Then, at each iteration, the new value of approximate solution for Φ^*n*+1^ is substituted into *V*^(*r*)^.

Additional numerical details as well as solution of toy models and error analysis are detailed in [24].

## Appendix B: Three species simulations

Our 3-species simulations added another component to the system–an algae. The algae donates fixed Carbon to *P vortex* and *E coli*, which effectively acts as additional nutrients. On the other hand, the algae compete with the bacteria on available nutrients.

Over all, our model is described by the following system of reaction-diffusion equations in which *X*(*x*,*y*,*t*) denotes the concentration of algae.

$$\begin{array}{l} \frac{\partial b_{1}}{\partial t}=\nabla \left[D_{1}b_{1}^{k}\nabla b_{1}\right]+\beta_{1}(n)(1+r_{X}X)b_{1}-\mu_{1}(n,a)b_{1}\\ \frac{\partial b_{2}}{\partial t}=\nabla \left[D_{2}\nabla b_{2}\right]+\nabla \left[C_{2}b_{2}\nabla b_{1}\right]+\beta_{2}(n)(1+r_{X}X)b_{2}-\mu_{2}(n)b_{2}\\ \frac{\partial X}{\partial t}=\nabla \left[D_{X}\nabla b_{2}\right]+\nabla \left[C_{X}b_{2}\nabla b_{1}\right]+\beta_{X}(n)X-\mu_{X}X\\ \frac{\partial n}{\partial t}=\nabla \left[D_{n}\nabla n\right]-\lambda_{1}(n)b_{1}-\lambda_{2}(n)b_{2}-\lambda_{X}(n)X\\ \frac{\partial a}{\partial t}=\nabla \left[D_{a}\nabla a\right]-p(a)b_{2} \end{array}$$ (6)

See Table 2 for simulation parameters.

**Table 2:** Parameter for 3-species simulations. Conversions between simulated and physical units are discussed in the results section.

Parameter	Value	Description
*r*_*X*_	1	Dependency coefficient of *P*. *vortex* reproduction by the algae
*D*_*X*_	0.25	Self-motility diffusion coefficient of the algae
*C*_*X*_	10	Advection coefficient of the algae with the flow of *P*. *vortex*
*β*_*X*_	0.25	Algae reproduction rate
*μ*_*X*_	0.1	Algae consumption rate
*λ*_*X*_	0.9	Nutrients consumption rate by the algae
*n*_0_	0.5	Initial concentration of *n*
*a*_0_	2	Initial concentration of *a*
${\bar{b}}_{\max}$	0.04	Maximal value for *b*_*1*_ at building state
${\bar{b}}_{\min}$	0.02	Minimal value for *b*_*1*_ at exploring state

## Supporting information

S1 FigParameters sensitivity analysis.A: *a*_0_ = 1, 3 (±50% of default value) B: *n*_0_ = 1, 3 (±50%); C1: ${\bar{b}}_{\max}=0.0396,\;0.0264\;(\pm 20\%)$; D: $C_{b_{2}}=4,\;8\;(\pm 50\%)$; E: $D_{b_{2}}=1.2E-4,\;8E-5\;(\pm 20\%)$.(TIF)

S2 Fig Simulations results with a modified rule for transitioning between builder/explorer phases. Results are practically the same as obtained with Eq (3). See the results section for details.
(TIF)
