# Supplementary figures and images for: Modeling cooperating micro-organisms in antibiotic environment
Source: PLoS One. 2017 Dec 28;12(12):e0190037. doi: 10.1371/journal.pone.0190037 (PMC5746235; doi:10.1371/journal.pone.0190037)

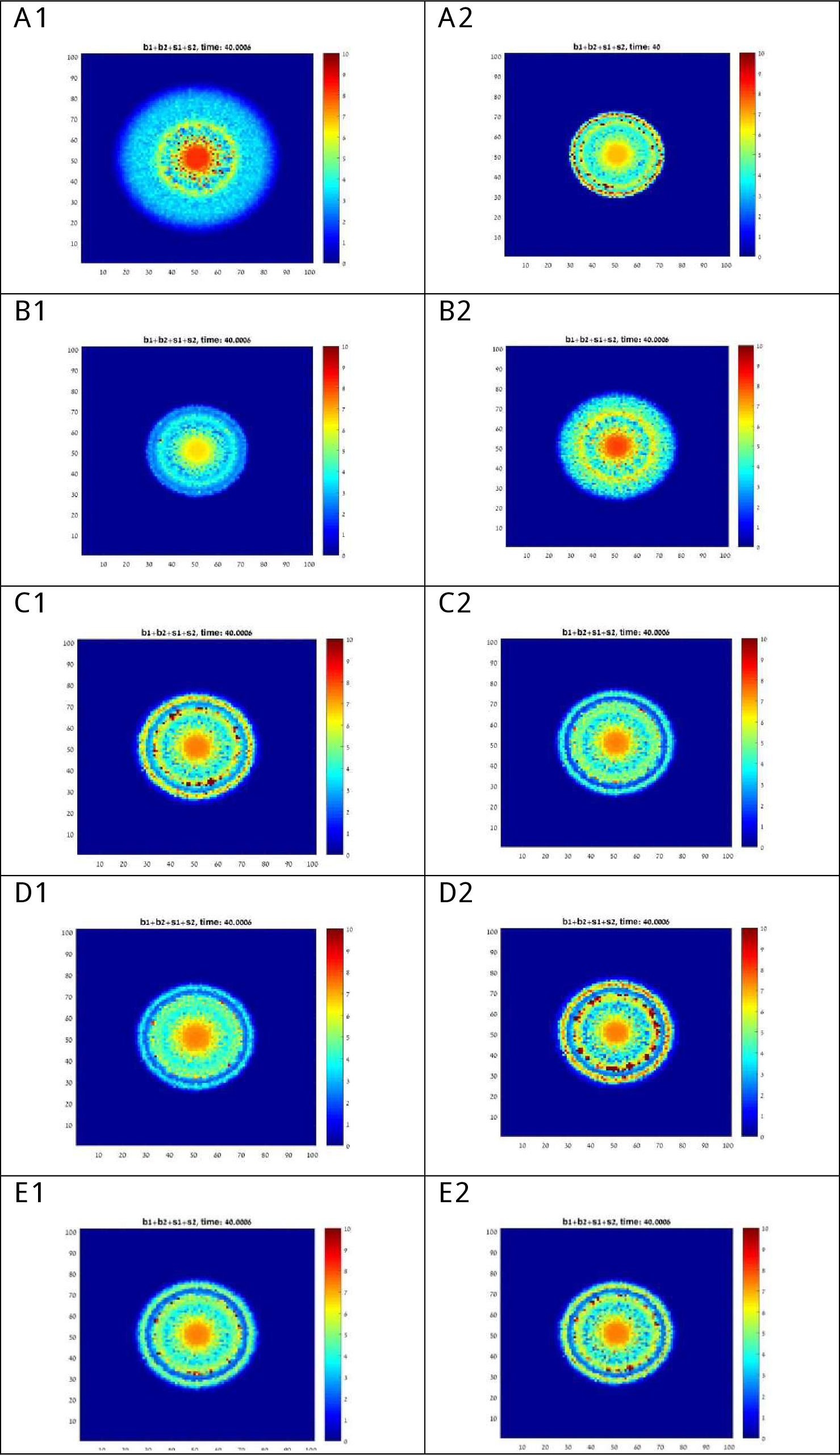

Supplement: S1 Fig — A: a0 = 1, 3 (±50% of default value) B: n0 = 1, 3 (±50%); C1: b¯max=0.0396,0.0264(±20%); D: Cb2=4,8(±50%); E: Db2=1.2E−4,8E−5(±20%). (TIF) [file pone.0190037.s001.tif]

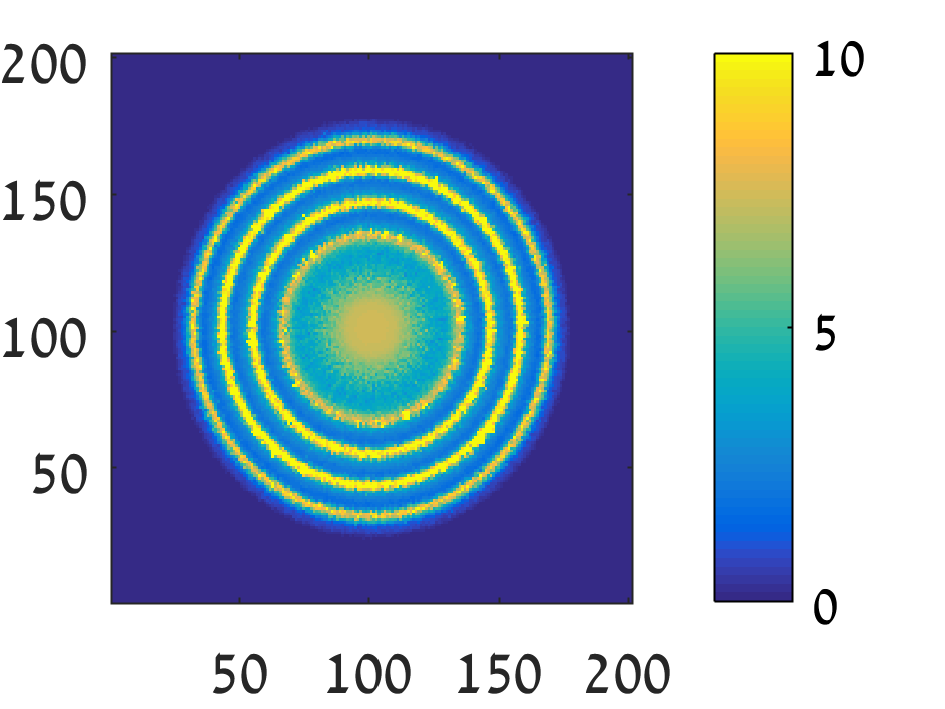

Supplement: S2 Fig — Results are practically the same as obtained with Eq (3). See the results section for details. (TIF) [file pone.0190037.s002.tif]
